# Medication preventing postictal hypoperfusion and cognitive side-effects in electroconvulsive therapy: A retrospective cohort study

**DOI:** 10.3389/fpsyt.2023.1026014

**Published:** 2023-02-09

**Authors:** Joey P. A. J. Verdijk, Gijsbert Schuur, Julia C. M. Pottkämper, Freek ten Doesschate, Jeannette Hofmeijer, Jeroen A. van Waarde

**Affiliations:** ^1^Department of Psychiatry, Rijnstate Hospital, Arnhem, Netherlands; ^2^Department of Clinical Neurophysiology, TechMed Centre, University of Twente, Enschede, Netherlands; ^3^Department of Neurology, Rijnstate Hospital, Arnhem, Netherlands

**Keywords:** electroconvulsive therapy, acetaminophen, non-steroidal anti-inflammatory drugs, calcium antagonists, cognitive outcome

## Abstract

**Background:**

Electroconvulsive therapy (ECT) is associated with postictal confusion and cognitive side-effects. In rats, acetaminophen, non-steroidal anti-inflammatory drugs (NSAIDs) and calcium antagonists decreased postictal cerebral hypoperfusion along with reduction in postictal symptoms. In this study, in ECT-patients, we explore associations between use of these potentially protective medications and occurrence of postictal confusion and cognitive outcome.

**Materials and methods:**

In this retrospective, naturalistic cohort study, patient-, treatment-, and ECT-characteristics, were collected from medical files of patients treated with ECT for major depressive disorder (MDD) or bipolar depressive episode. To test for associations of use of these medications with occurrence of postictal confusion, 295 patients could be included. Cognitive outcome data were available in a subset of 109 patients. Univariate analyses and multivariate censored regression models were used to test for associations.

**Results:**

Occurrence of severe postictal confusion was not associated with use of acetaminophen, NSAIDs or calcium antagonists (*n* = 295). Regarding the cognitive outcome measure (*n* = 109), use of calcium antagonists was associated with higher post-ECT cognitive scores (i.e., better cognitive outcome; β = 2.23; *p* = 0.047), adjusted for age (β = −0.02; *p* = 0.23), sex (β = −0.21; *p* = 0.73), pre-ECT cognitive score (β = 0.47; *p* < 0.0001), and post-ECT depression score (β = −0.02; *p* = 0.62), but use of acetaminophen (β = −1.55; *p* = 0.07) as well as NSAIDs (β = −1.02; *p* = 0.23) showed no associations.

**Conclusion:**

This retrospective study does not find arguments for protective effects of acetaminophen, NSAIDs or calcium antagonists against severe postictal confusion in ECT. As a preliminary finding, the use of calcium antagonists was associated with improved cognitive outcome after ECT in this cohort. Prospective controlled studies are necessary.

## Introduction

Electroconvulsive therapy (ECT) is an effective and safe treatment option for some severe psychiatric disorders. Unfortunately, ECT is associated with cognitive side-effects which hamper more broad application and successful treatment (1). About one third of patients may experience postictal confusion immediately after the ECT-sessions, which may cause discomfort and even premature termination of this potentially beneficial treatment (2). A quarter of ECT-patients show a significant decrease in cognitive functioning 1 week after cessation of the ECT-course (3). These cognitive impairments may restore within 3 months, although this was only studied at group-level (4). Though, the within-group variability of cognitive functioning after ECT is large and some patients do experience long-term cognitive impairment. Interventions that reduce postictal confusion and cognitive impairment would, therefore, be of great relevance to the clinical practice of ECT.

Several studies have addressed postictal confusion and cognitive side-effects of ECT (5, 6). Clinical research has not provided robust predictors for the occurrence of postictal confusion (7, 8). Age, pre-existent cognitive problems, used electrode placement, number of administered ECT-sessions, and persisting depressive symptoms after ECT were described as determinants of cognitive outcome of ECT. However, a recent systematic review and meta-analysis only identified a younger age as factor related to cognitive deterioration after the ECT-course (5). The literature reports several pathophysiological theories regarding negative cognitive ECT-outcome, including changes in neuroinflammation, neurotrophic factors, and hormones (9). Magnetic resonance imaging (MRI) studies have shown an increase in hippocampal volume associated with increased cognitive disturbances (5). Other studies have found functional brain connectivity (fMRI) changes in the salience-, frontoparietal-, and default mode-networks which correlated with cognitive changes (9). Regarding preventive strategies to diminish cognitive side-effects, a comprehensive meta-analysis has found evidence for protective effects of liothyronine and memantine in ECT, underlining the divergent hypotheses regarding the pathophysiological mechanisms causing postictal confusion and cognitive deterioration after ECT (10).

A new and plausible hypothesis has recently been proposed from animal studies on postictal cerebral hypoperfusion (11, 12). In rats, a clear decrease in postictal cerebral perfusion was measured after electrically evoked seizures, which compared with postictal hypoperfusion in humans with spontaneous seizures. Moreover, in the animal model, the magnitude of cerebral hypoperfusion was associated with the severity of postictal symptoms (i.e., paralysis and amnesia). Also, in subsequent experiments, it was shown that administration of acetaminophen, non-steroidal anti-inflammatory drugs (NSAIDs) and calcium antagonists decreased postictal cerebral hypoperfusion along with a decrease in postictal symptoms (11). These preventive effects may be explained as follows. Physiologically, cerebral perfusion is governed by local prostaglandins in the brain vasculature. Acetaminophen and NSAIDs, which are regularly used medications, block the prostaglandin pathway through inhibition of cyclooxygenase (COX)-2. Calcium antagonists influence the diameter of the vasculature more directly, by causing relaxation of vascular smooth muscle around blood vessels (13). Indeed, in the animal model, acetaminophen, NSAIDs and calcium antagonists were effective in ameliorating postictal hypoperfusion and postictal symptoms, if given hours before the seizure induction (11). Moreover, calcium antagonists also showed effectiveness when administered after the seizure (11). Until now, no clinical studies in patients have explored the potentially protective effects of these medications to prevent postictal confusion and cognitive side-effects after ECT.

Here, we aim to examine the associations between the use of acetaminophen, NSAIDs or calcium antagonists and (1) the occurrence of severe postictal confusion directly after ECT-sessions and (2) the cognitive outcome after completed ECT-courses. We hypothesize that the use of (any of) these potentially protective medications is associated with lower occurrence of severe postictal confusion and less cognitive deterioration after the ECT-course.

## Materials and methods

### Design

We performed a hypothesis-driven, retrospective, naturalistic cohort study of patients treated with ECT at Rijnstate Depression Center of the Department of Psychiatry, Rijnstate Hospital, Arnhem, The Netherlands. All data were collected from patients’ medical records. In Rijnstate, approximately 1,400 ECT-sessions take place annually, mostly to treat major depressive episodes as part of major depressive disorder (MDD) and bipolar disorder (BD).

### Participants

Patients were eligible for inclusion if they (1) were treated with at least four ECT-sessions in the period of December 2009 until December 2020; (2) had completed their ECT-course; and (3) had a complete medical record. For the outcome variable “severe postictal confusion,” only data of patients having electronic medical records were included, since postictal confusion was not systematically registered in the paper medical records. To evaluate associations with cognitive outcome after the ECT-course, patients were excluded if no validated cognitive scales were available (measured within 1 month before and within 1 month after the course). Furthermore, because we intended to adjust our analyses for post-ECT depression severity, only patients treated for MDD or BD were included. Data were stored and analyzed anonymously. The Central Committee on Research Involving Human Subject waived the need for informed consent of patients (CCMO-number: 2020-6879).

### Electroconvulsive therapy

Electroconvulsive therapy was performed according to the Dutch treatment guidelines (14), using a Thymatron System IV (Somatics Incorporation, Lake Bluff, IL, USA). A constant current (900 mA), brief pulse (0.25–1.0 ms) was used. Electrode position [i.e., right unilateral according to D’Elia (RUL), bifrontotemporal (BL) or left unilateral (LUL)] was chosen by the treating psychiatrist depending on the patients’ clinical condition. The initial dose was determined by titration (i.e., 2.5 seizure threshold in BL and six times seizure threshold in RUL/LUL ECT) or by age-based methods. The standard sedative agent was etomidate (0.2–0.3 mg/kg body mass) and succinylcholine (0.5–1.0 mg/kg body mass) was used as a muscle relaxant.

### Outcome measures

We defined two main outcome measures, namely, (1) occurrence of severe postictal confusion directly after any ECT-session and (2) cognitive change after the completed ECT-course. First, we determined the frequency of occurrence of severe postictal confusion, which we defined as disturbing behavior of the patient within 4 h after an ECT-session that required direct medical interventions (i.e., administration of additional sedatives, application of constraints, and/or other restrictions) without report of other medical reasons for this acute change in behavior. Second, we established cognitive change after the completed ECT-course with the use of validated cognitive tests. Cognitive change was calculated by subtracting baseline scores [measured within 1 month before treatment with mini-mental state examination (MMSE) (15) or Montreal Cognitive Assessment (MoCA) (16)] from the end-scores measured within 1 month after the ECT-course. MoCA-scores were converted to MMSE-equivalents (17).

### Determinants

We scrutinized medical records for the main determinants of interest, namely, concomitant use of acetaminophen, NSAIDs and calcium antagonists. Also, we extracted possible confounding covariates, namely, demographic variables (i.e., sex and age), clinical variables (i.e., primary psychiatric diagnosis, nicotine and alcohol use, measures on mood severity scales) and ECT-parameters (i.e., electrode position applied at the end of the ECT-course, total number of ECT-sessions during the course). Mood scores were included if measured within 1 month before starting ECT with the Montgomery–Åsberg Depression Rating Scale [MADRS, (18)] or the Hamilton Depression Rating Scale [HDRS, (19)], as well as measured within 1 month after the ECT-course. MADRS-scores were converted to HDRS-equivalents (20).

### Univariate analyses

Baseline variables were described as frequencies and percentages for dichotomous variables, means ± standard deviation (SD) for normally distributed continuous variables, and medians and interquartile ranges (IQRs) for non-normally distributed continues variables. To examine our first outcome measure, patients with and without occurrence of severe postictal confusion were compared, including the use of (any) potentially protective medications, by using chi square tests for categorical variables, independent-samples *t*-tests if assumptions were met, or Wilcoxon rank-sum tests. Before analyzing our second outcome measure (i.e., cognitive change during the ECT-course), we calculated the *reliable change index* [RCI (21)], which indicates whether a change in score between baseline and follow-up MMSE was significantly larger than expected based on test-retest reliability. Therefore, a 90% confidence interval (i.e., alpha = 0.10) was used, which translates to interpreting RCI values greater than 1.645 times SD as reliable (3). We used the calculation formula as proposed by Jacobson and Truax (22), executed in the R package “JTRCI” (23): RCI = (post-test–pre-test)/Standard error of the difference (SED). The SED was calculated by using the SD of baseline scores and the reliability measure of the MMSE from the literature (24). After this step, patients that reliably cognitively deteriorated were compared with those who showed no reliable change and those who reliably cognitively improved by using Fisher’s exact tests for categorical variables and ANOVA’s or Kruskal–Wallis tests for continuous variables.

### Multivariate analyses

In our multivariate analyses, we tested for associations between the use of the potentially protective medications with (1) occurrence of postictal confusion and (2) cognitive change after ECT, corrected for known confounding covariates. As covariates in all multivariate models, we included age, sex, number of ECT-sessions, and bilateral electrode position (5). Additionally to these, we added two extra covariates in the models for cognitive outcome, namely, post-ECT HDRS-score (because residual depressive symptoms after the ECT-course might confound cognitive functioning) and pre-ECT MMSE-score (to correct for baseline cognitive functioning).

To examine our first outcome measure (i.e., multivariate associations with occurrence of severe postictal confusion), we applied two binominal logistic regression analyses [General Linear Model (GLM)]. In the first, “occurrence of postictal confusion” (dichotomous) was used as a dependent variable and “any use of acetaminophen, NSAIDs or calcium antagonists” (dichotomous) and the confounding covariates as independent variables. In the second GLM, to test uniquely explained variance by each of the medications, “occurrence of postictal confusion” (dichotomous) was used as a dependent variable and, separately, “use of acetaminophen,” “use of NSAIDs,” and “use of calcium antagonists” (dichotomous) were used as independent variables together with the confounding covariates.

To examine our second outcome measure (i.e., multivariate associations with cognitive outcome), we applied two censored regression models (25), because our cognitive outcome parameters showed ceiling effects (i.e., a large number of measurements reached the maximum MMSE- score of 30, leading to considerate heteroskedasticity). In the first model, the post-ECT MMSE-score was used as dependent variable, and “any use of acetaminophen, NSAIDs or calcium antagonists” (dichotomous) with the confounding covariates as independent variables. In the second model, the post-ECT MMSE-score was used as dependent variable and, separately, “use of acetaminophen,” “use of NSAIDs,” or “use of calcium antagonists” (dichotomous) were used as independent variables together with the confounding covariates.

Before evaluating the results of our regression analyses, the assumption of linearity of the logit was tested by inputting the logit of the predictors in the regression models and checking for significant interactions. Also, collinearity was tested by calculating variance inflation factors (VIFs) and checking if these were <10. All statistical analyses were performed by using R version 4.1.1 [including add-on R package “censReg” (26)] and associations were quantified with the use of odds ratios (OR), where possible. *P*-values ≤ 0.05 were regarded as statistically significant.

## Results

**FIGURE 1:**
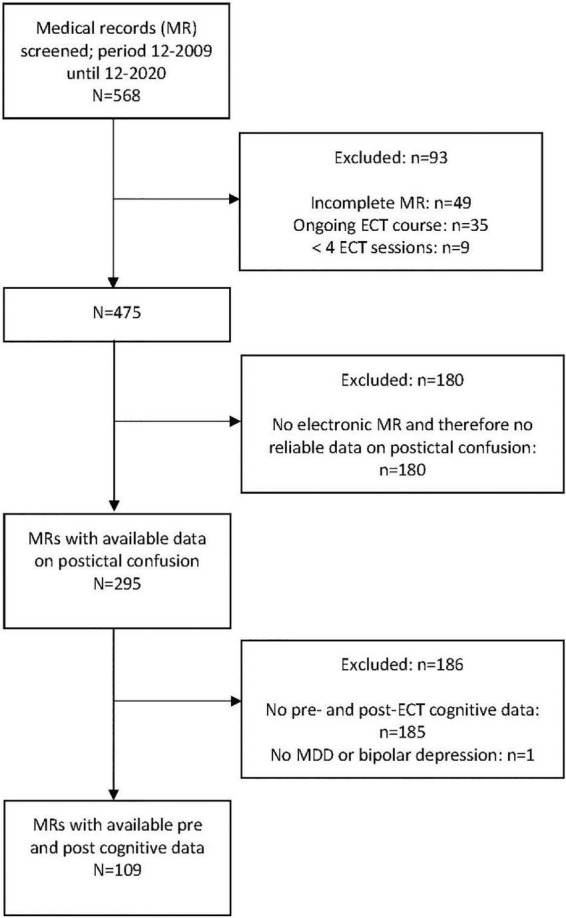
Flowchart of patient inclusion.

From 2009 to 2020, in total 568 patients were screened for inclusion of whom 475 met our inclusion criteria. Of these, 295 patients’ files had complete data regarding the first outcome measure of occurrence of severe postictal confusion and could be included in our analyses. For our second outcome measure, additional cognitive outcome data were available from 109 patients (Figure 1). Patient-, treatment-, and outcome-characteristics of the included sample are presented in Table 1. Patients’ age ranged from 16 to 91 years (mean = 57.3 ± 15.5 SD years) and 64% (*n* = 189) was female. Most patients were treated with ECT for MDD (*n* = 209; 71%). Median mood score decreased significantly after the ECT-course (*p* < 0.001).

**TABLE 1:** Patient-, treatment-, and outcome characteristics in 295 patients who were treated with electroconvulsive therapy (ECT).

	Patients with available data (*n*)	*n* (%), median (IQR^a^), mean ± SD^b^
**Demographic variables**		
Age, mean ± SD years	295	57.3 ± 15.5
Female sex [*n* (%)]	295	189 (64.0)
Nicotine use	295	79 (26.8)
Alcohol use	295	61 (20.7)
Drug use	295	6 (2.0)
**Psychiatric diagnosis/Indication for ECT**		
Major depressive disorder [*n* (%)]	295	209 (71)
Bipolar depressive episode [*n* (%)]	295	45 (15.3)
Schizoaffective disorder [*n* (%)]	295	16 (5.4)
Schizophrenia [*n* (%)]	295	1 (0.3)
Other psychosis [*n* (%)]	295	5 (1.7)
Catatonia [*n* (%)]	295	19 (6.4)
**ECT variables**		
Total number of ECT-sessions, mean ± SD	295	16.9 ± 9.4
Bilateral electrode position at end of ECT-course [*n* (%)]	295	235 (79.7)
**Treatment outcome in HDRS^c^ equivalents**		
Baseline mood score, median (IQR)	107	26.0 (11)^e^
Mood after ECT course, median (IQR)	105	11.0 (11)^e^
Change in mood score, mean ± SD	105	−14.4 ± 9.9
**Cognitive outcome in MMSE^d^ equivalents**		
Baseline cognition score, median (IQR)	109	29.0 (4)^f^
Cognition score after ECT-course, median (IQR)	109	29.0 (3)^f^
Change in cognition score, mean ± SD	109	0.5 ± 3.2
Occurrence of severe postictal confusion directly after any ECT-session [*n* (%)]	295	74 (25)

^a^Interquartile range.

^b^standard deviation.

^c^Hamilton Depression Rating Scale.

^d^mini-mental state examination.

^e^Wilcoxon Signed Rank test, *p* < 0.05.

^f^Wilcoxon Signed Rank test, *p* < 0.05.

### Associations between use of potentially protective medication and occurrence of postictal confusion

Out of 295 ECT-patients, 74 (25%) showed severe postictal confusion. In the univariate analyses, none of the variables differed between patients with or without occurrence of postictal confusion (see Table 2). Furthermore, binominal logistic regression analyses showed no associations between occurrence of postictal confusion and any use of the medications [β = 0.14, *p* = 0.65, OR = 1.15 (95% CI = 0.6–2.1)], the use of acetaminophen [β = 0.20, *p* = 0.55, OR = 1.22 (95% CI = 0.62–2.33)], the use of NSAIDs [β = −0.04, *p* = 0.96, OR = 0.98 (95% CI = 0.37–2.23)], or the use of calcium antagonists [β = 0.01, *p* = 0.85, OR = 1.10 (95% CI = 0.37–2.90)], adjusted for the selected covariates. Only female sex was independently associated with a lower occurrence of postictal confusion [β = −0.54, *p* = 0.049; OR = 0.58 (95% CI = 0.3–1.0)].

**TABLE 2:** Univariate statistics of patient-, treatment-, and outcome characteristics in 295 patients treated with electroconvulsive therapy (ECT), in whom severe postictal confusion occurred directly after any ECT-session (*n* = 74) and in those without sPIC (*n* = 221).

	Occurrence of severe postictal confusion	Without occurrence of severe postictal confusion	
Variables	*n* = 74	*n* = 221	Significance
**Demographic variables**			
Age in years, mean ± SD	57.4 (12.8)	57.3 (16.4)	0.95^a^
Female sex [*n* (%)]	40 (21.2)	149 (78.8)	0.053^b^
Nicotine use [*n* (%)]	24 (32.4)	55 (24.9)	0.10^b^
Alcohol use [*n* (%)]	18 (24.3)	43 (19.5)	0.27^b^
Drug use [*n* (%)]	1 (1.4)	5 (2.3)	0.63^c^
Major depressive disorder [*n* (%)]	52 (70.3)	157 (71.0)	1.0^b^
Bipolar depressive episode [*n* (%)]	15 (20.3)	30 (13.6)	0.23^b^
Schizoaffective disorder [*n* (%)]	4 (5.4)	12 (5.4)	1.0^b^
Schizophrenia [*n* (%)]	0 (0)	1 (0.5)	1.0^c^
Other psychosis [*n* (%)]	0 (0)	5 (2.2)	0.34^c^
Catatonia [*n* (%)]	3 (4.0)	16 (7.2)	0.25^c^
**ECT variables**			
Total number of ECT-sessions during course, mean^d^ ± SD	17.3 (11.1)	16.8 (8.8)	0.75^e^
Bilateral electrode position at end of ECT-course [*n* (%)]	59 (79.7)	176 (79.6)	1.0^b^
**Use of protective medications present during ECT-course**			
Any protective medications [*n* (%)]	25 (34.2)	71 (32.1)	0.85^b^
Acetaminophen [*n* (%)]	17 (23.3)	47 (21.3)	0.84^b^
Used dosage of acetaminophen in users in mg, mean (SD)	2,117.7 (1,166)	2,372.3 (1,373)	0.60^e^
Non-steroid anti-inflammatory drugs [*n* (%)]	8 (11.0)	23 (10.4)	1.0^b^
Calcium antagonists [*n* (%)]	6 (8.2)	16 (7.2)	0.98^b^
Triptans [*n* (%)]	0	0	N/A
**Depression treatment outcome in available HDRS equivalents^f^**			
Baseline mood score, median (IQR), *n* = 139	26 (8)	25 (9)	0.88^e^
Mood after ECT-course, median (IQR), *n* = 104	12 (10)	11 (9)	0.38^e^
Change in mood score, mean ± SD, *n* = 42	−12 (13.1)	−10.9 (8.4)	0.75^a^
**Cognition outcome in available MMSE equivalents^f^**			
Baseline cognition score, median (IQR), *n* = 66	29 (2.5)	29 (4)	0.12^e^
Cognition score after ECT-course, median (IQR), *n* = 59	29 (3)	28 (3.75)	0.14^e^
Change in cognition score, mean ± SD, *n* = 33	0.25 (2.4)	−0.08 (1.6)	0.65^a^

^a^Independent-sample *t*-test.

^b^Chi square test.

^c^Fisher’s exact test.

^d^Even though distribution not normal, means are displayed, since medians are 0 (0).

^e^Wilcoxon rank-sum test.

^f^Data of both outcome measures were only included if scores were available within 4 weeks before and within 4 weeks after the ECT-course.

### Associations between use of potentially protective medication and cognitive change after ECT

**FIGURE 2:**
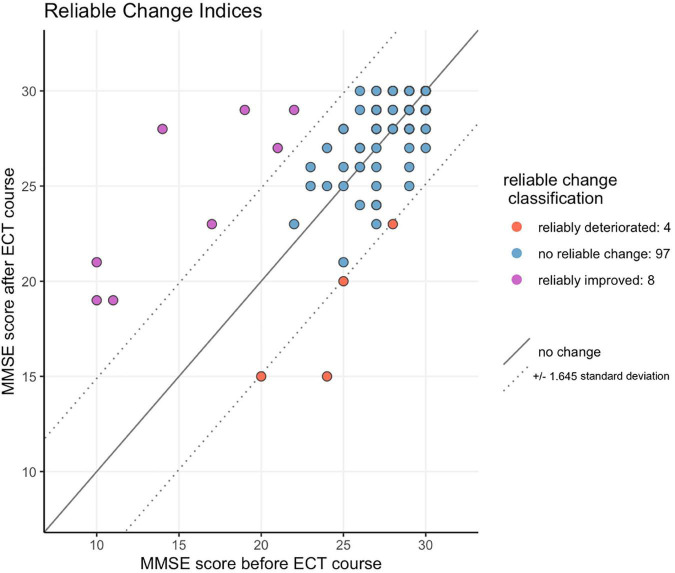
Reliable change in cognition score after the ECT-course compared to baseline.

By using the RCI, out of 109 patients, only 12 (11.0%) showed a reliable change in cognitive score after the ECT-course compared to baseline: 4 (3.7%) patients cognitively deteriorated, 97 (90%) patients remained unchanged, and 8 (7.3%) patients improved (Figure 2). Table 3 summarizes the univariate comparisons between these three cognitive outcome groups. Patients with reliable cognitive improvement (*n* = 8) were significantly older than those who did not cognitively change (87.6 ± 7.6 SD years vs. 55.9 ± 15.3 SD years, respectively; *p* < 0.001). Mean age of patients with reliable cognitive deterioration did not differ from that of patients with no cognitive change (62.5 ± 15.0 SD years vs. 55.9 ± 15.3 SD years, respectively; *p* = 0.38). Nicotine use was significantly higher in patients with no reliable cognitive change (38.1%) compared to those who reliably improved (0%) or deteriorate (0%).

**TABLE 3:** Univariate statistics of patient-, treatment-, and outcome characteristics in 109 patients, who reliably cognitive deteriorated (*n* = 4), reliably cognitive unchanged (*n* = 97), and reliably cognitive improved (*n* = 8) after electroconvulsive therapy (ECT).

	Cognitive functioning reliably deteriorated after ECT-course	Cognitive functioning was reliably unchanged after ECT-course	Cognitive functioning reliably improved after ECT-course	
Variables	*n* = 4	*n* = 97	*n* = 8	Significance
**Demographic variables**				
Age in years, mean ± SD	62.50 (15.0)	55.85 (15.3)	78.62 (7.60)	0.0003^a,b^
Female sex [*n* (%)]	3 (75.0)	60 (61.9)	6 (75.0)	0.79^c^
Nicotine use [*n* (%)]	0 (0)	37 (38.1)	0 (0)	0.03^c^
Alcohol use [*n* (%)]	0 (0)	29 (29.9)	0 (0)	0.10^c^
Drug use [*n* (%)]	0 (0)	1 (1.0)	0 (0)	1.0^c^
**ECT variables**				
Number of ECT-sessions during course, median (IQR)	18.0 (4.75)	18.0 (10)	17.0 (10)	0.96^d^
Bilateral electrode position at end ECT-course [*n* (%)]	3 (75.0)	63 (64.9)	8 (100)	0.11^c^
**Depression treatment outcome in HDRS equivalents^e^**				
Baseline mood score, median (IQR)	30 (12.75)	26 (11)	30.5 (7.5)	0.29^d^
Mood after ECT-course, median (IQR)	15.5 (14.25)	12 (11)	10.5 (5.75)	0.85^d^
Change in mood score, mean ± SD	−14.5 (18.5)	−14.1 (9.5)	−17.62 (10.0)	0.63^a^
**Cognitive outcome in MMSE^f^ equivalents**				
Baseline cognition score, median (IQR)	24.3 (2.75)	29 (3)	15.5 (8.75)	0.29^d^
Cognition after ECT-course, median (IQR)	17.5 (5.75)	29 (2)	25 (7.75)	0.85^d^
Change in cognition score, mean ± SD	−6 (2)	0.1 (1.6)	8.9 (2.8)	7.2e–08^d^
**Use of protective medications during ECT-course**				
Any protective medications [*n* (%)]	3 (75.0)	23 (23.7)	7 (87.5)	0.0001^c, g^
Acetaminophen [*n* (%)]	2 (50.0)	11 (11.3)	3 (37.5)	0.02^c,h^
Non-steroid anti-inflammatory drugs [*n* (%)]	2 (50)	13 (13.4)	5 (62.5)	0.002^c,i^
Calcium antagonists [*n* (%)]	0 (0)	7 (7.2)	3 (37.5)	0.045^c,g^
Triptans [*n* (%)]	0 (0)	0 (0)	0 (0)	N/A

^a^ANOVA.

^b^*Post hoc* Bonferroni shows significant difference between reliably improved and unchanged group, but not in the other comparisons.

^c^Fisher’s exact test.

^d^Kruskal–Wallis.

^e^Hamilton Depression Rating Scale.

^f^Mini-mental state examination.

^h^Pairwise Fisher’s exact does not show pairwise significant differences.

^g^Pairwise Fisher’s exact shows significant difference between reliably improved and unchanged group (*p* = 0.0006), but not between reliably deteriorated and unchanged.

^i^Pairwise Fisher’s exact shows a significant difference between reliably improved and no reliable change (*p* = 0.004), but not between reliably deteriorated and unchanged.

**FIGURE 3:**
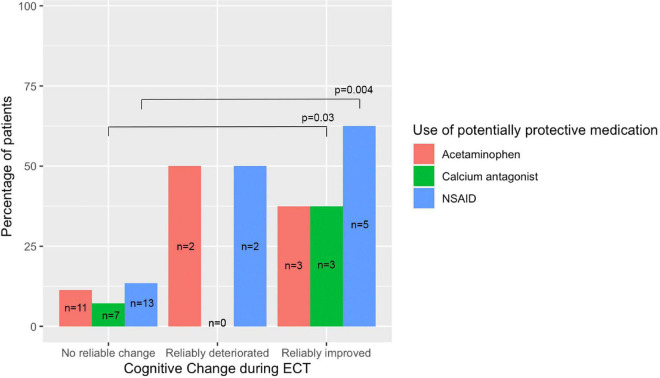
Frequencies of the use of potentially protective medications in the three cognitive outcome groups. Only the statistically significant comparisons (pairwise Fisher’s exact) are depicted.

Figure 3 summarizes the frequencies of the use of the potentially protective medications in the three cognitive outcome groups. Only statistically significant comparisons (pairwise Fisher’s exact) are depicted. In the univariate analyses, any use of potentially protecting medications was associated with reliable cognitive improvement [*n* = 7 (87.5%) vs. *n* = 23 (23.7%); pairwise Fisher’s exact; *p* < 0.001], but also with reliable cognitive deterioration [*n* = 3 (75%) vs. *n* = 23 (23.7%); pairwise Fisher’s exact; *p* = 0.051].

Although subsample sizes were low, frequencies and percentages of patients in the three reliable cognitive outcome groups were calculated for (non-)users of acetaminophen, NSAIDs or calcium antagonists separately (see Table 3). Reliable cognitive improvement was found in three acetaminophen users, in five NSAIDs users and in three calcium antagonists’ users. Reliable cognitive deterioration was found in two acetaminophen users, in two NSAIDs users and in none of the calcium antagonists’ users. No cognitive change was found in 11 acetaminophen users, in 12 NSAIDs users and in 12 calcium antagonists’ users. Results of statistical tests comparing between these groups are provided in Table 3, but although Fisher’s exact tests were used for low numbers these should be interpreted with caution.

Censored multivariate analysis showed no association of post-ECT MMSE-score with any use of acetaminophen, NSAIDs or calcium antagonists combined (β = −0.94; *p* = 0.19), adjusted for age (β = −0.02; *p* = 0.33), sex (β = −0.10; *p* = 0.87), electrode placement (β = −0.73; *p* = 0.33), number of ECT-sessions (β = 0.02; *p* = 0.62), pre-ECT MMSE-score (β = 0.48; *p* < 0.0001), and post-ECT HDRS-score (β = −0.03; *p* = 0.51). When examining the individual medications, censored multivariate analysis showed that use of calcium antagonists was associated with higher post-ECT MMSE-scores (i.e., better cognitive outcome; β = 2.23; *p* = 0.047), adjusted for age (β = −0.02; *p* = 0.23), sex (β = −0.21; *p* = 0.73), pre-ECT MMSE-score (β = 0.47; *p* < 0.0001), and post-ECT HDRS-score (β = −0.02; *p* = 0.62). Adjusting for the same covariates, use of acetaminophen (β = −1.55; *p* = 0.07) as well as NSAIDs (β = −1.02; *p* = 0.23) showed no associations with post-ECT MMSE-scores.

## Discussion

This is–to our knowledge–the first study exploring associations of concomitant use of acetaminophen, NSAIDs or calcium antagonists in ECT-patients with (1) the occurrence of severe postictal confusion and (2) cognitive outcome. Based on experimental animal studies, we hypothesized that these medications may prevent or reduce postictal hypoperfusion and subsequently reduce the risk of postictal confusion and cognitive side-effects. Regarding occurrence of postictal confusion, we found no associations with the use of acetaminophen, NSAIDs or calcium antagonists. Interestingly, we found a significant association of the concomitant use of calcium antagonists with improved cognitive outcome after the ECT-course, but not of use of acetaminophen and NSAIDs.

### Possible protective potency of concomitant use of calcium antagonists on cognitive outcome

Concomitant use of calcium antagonists was found to be associated with improved cognitive outcome after ECT. Although this may compare with findings in animal models (11), interpretation of this finding must be done with caution because this isolated association was only established in our multivariate model (*n* = 109). In contrast to calcium antagonists, the concomitant use of acetaminophen and NSAIDs were not associated with cognitive outcome. We hypothesize that this contrast may be due to a more direct effect of calcium antagonists on the cerebral vasculature compared to acetaminophen and NSAIDs. Calcium antagonists exert their effect by directly blocking the calcium channels in vascular smooth muscle, leading to vasodilatation (13). In contrast, acetaminophen and NSAIDs act earlier in the hypothesized cascade of cerebral vasoconstriction and additional pathways may be involved in regulating cerebral blood flow (11). Interestingly, in the animal model, calcium antagonists were found to be effective even when administered directly after the seizure, further strengthening the hypothesis of a more direct influence of calcium antagonists in comparison to acetaminophen and NSAIDs. Calcium antagonists may thus be more effective in preventing postictal hypoperfusion and improving cognitive outcome than acetaminophen or NSAIDs.

Again, caution in the interpretation of these preliminary results is needed, because most users of calcium antagonists (as well as users of acetaminophen and NSAIDs) did not show a reliable cognitive change after the ECT-course. Possibly, the limited number of cognitively improved (*n* = 8) and deteriorated (*n* = 4) patients in our sample may explain the conflicting results in the univariate analyses. Therefore, in the multivariate regression models, we used the continuous score of cognitive outcome (*n* = 109). We cannot exclude the possibility of interactions with other confounding covariates. ECT-patients with different physical comorbidities, who used acetaminophen, NSAIDs or calcium antagonists for these comorbidities, might have confounded the cognitive outcome. Therefore, our explorative and preliminary findings deserve further prospective, randomized controlled studies, of which one is in progress (NCT04028596) (27).

### Strengths and limitations

This study has several strengths. We included a large naturalistic cohort of ECT-patients from a period of 11 years. Our results reflect *real world* ECT-practice and might be directly translated to clinical practice. However, multiple limitations reduce the strength of our conclusions. First, the observational design of the study negates conclusions of causality. Second, even though we tried to control for different possible confounding variables, we cannot exclude that other (unknown) confounders have influenced our results. For example, we did not include the indications for concomitant use of acetaminophen, NSAIDs and calcium antagonists (e.g., cardiovascular and rheumatological illnesses) in our analyses, while these comorbidities are probably associated with (more) use of antihypertensive medications and analgesics. Third, we used a definition of a relatively severe occurrence of postictal confusion (7). Therefore, our results cannot be extrapolated to more subtle forms of postictal confusion. Fourth, we included only cognitive screening tests (i.e., MMSE and MoCA) as cognitive outcome measure. These instruments may be unable to capture subtle changes due to ceiling effects, especially in younger patients (3). This may have led to an underrepresentation of younger patients in the reliably changed cognitive score groups. Also, the timing of cognitive outcome measurement ranged significantly (from a few days after the last ECT-session until long-term effects after 4 weeks). Fifth, we were unable to extract the exact timing of the daily administration of the relevant medications in relation to the ECT-sessions. Sixth, even though the sample size of patients with cognitive measures was considerable (*n* = 109), only a small number of patients (*n* = 12, 11%) showed a reliable cognitive change, which has limited the power of our analyses.

In conclusion, this retrospective, hypothesis-driven cohort study did not show arguments for acetaminophen, NSAIDs or calcium antagonists to prevent severe postictal confusion directly after the ECT-sessions. As a preliminary finding, concomitant use of calcium antagonists was associated with better cognitive outcome after the ECT-course, but not the use of acetaminophen and NSAIDs. Prospective controlled studies are needed for definite conclusions regarding the potency of acetaminophen, NSAIDs or calcium antagonists to reduce postictal hypoperfusion, severe postictal confusion and cognitive side-effects in ECT-patients.

## Data availability statement

The raw data supporting the conclusions of this article will be made available by the authors, without undue reservation.

## Ethics statement

Ethical review and approval was not required for the study on human participants in accordance with the local legislation and institutional requirements. Written informed consent for participation was not required for this study in accordance with the national legislation and the institutional requirements.

## Author contributions

GS, JV, and JW designed the study concept. GS and JV performed the data collection. JV, JP, and FD performed data analysis. JW and JH supervised the study. All authors drafted the manuscript and had full responsibility for the decision to submit for publication.
